# Morquio B Disease. Disease Characteristics and Treatment Options of a Distinct *GLB1*-Related Dysostosis Multiplex

**DOI:** 10.3390/ijms21239121

**Published:** 2020-11-30

**Authors:** Nataliya Yuskiv, Katsumi Higaki, Sylvia Stockler-Ipsiroglu

**Affiliations:** 1 BC Children’s Hospital, University of British Columbia, Vancouver, BC V6H 3V4, Canada; nyuskiv@cw.bc.ca; 2 Research Initiative Center, Organization for Research Initiative and Promotion, Tottori University, Yonago 683-8503, Japan; kh4060@med.tottori-u.ac.jp

**Keywords:** Mucopolysaccharidosis type 4, MPS4B, GM1 gangliosidosis, beta-galactosidase, *GLB1*, keratan sulfate, dysostosis multiplex, spondylo-epiphyseal dysplasia, dystonia, developmental delay

## Abstract

Morquio B disease (MBD) is an autosomal recessive *GLB1*-gene-related lysosomal storage disease, presenting with a peculiar type of dysostosis multiplex which is also observed in *GALNS*-related Morquio A disease. MBD may present as pure skeletal phenotype (*pure MBD*) or in combination with the neuronopathic manifestations seen in type 2 (juvenile) or type 3 (late onset) GM1 gangliosidosis (*MBD plus*). The main skeletal features are progressive growth impairment, kyphoscoliosis, coxa/genua valga, joint laxity, platyspondyly and odontoid hypoplasia. The main neuronopathic features are dystonia, ataxia, and intellectual/developmental/speech delay. Spinal cord compression occurs as a complication of spinal dysostosis. Chronic pain is reported, along with mobility issues and challenges with daily living and self-care activities, as the most common health concern. The most commonly reported orthopedic surgeries are hip and knee replacements. Keratan sulphate-derived oligosaccharides are characteristic biomarkers. Residual β-galactosidase activities measured against synthetic substrates do not correlate with the phenotype. W273 L and T500A are the most frequently observed *GLB1* variants in MBD, W273L being invariably associated with *pure MBD*. Cytokines play a role in joint destruction and pain, providing a promising treatment target. In the future, patients may benefit from small molecule therapies, and gene and enzyme replacement therapies, which are currently being developed for GM1 gangliosidosis.

## 1. Introduction

Morquio B disease (MBD), also called Mucopolysaccharidosis IVB (OMIM # 253010), is a rare lysosomal storage disorder with a reported prevalence of 1:250,000–1,000,000 live births [1]. Genetically, MBD is caused by distinct mutations in the *GLB1* gene. *GLB1* mutations are typically associated with a progressive neuronopathic condition spanning from infantile to juvenile and late onset GM1 gangliosidosis. Yet MBD (OMIM # 230500) presents with a unique dysostosis multiplex, causing a peculiar type of spondylo-epiphyseal dysplasia with or without additional neuronopathic manifestations. While genetically MBD is an allelic variant of GM1 gangliosidosis, clinically it is a mild phenocopy of *GALNS*-related Morquio A disease, which is characterized by the same type of spondyloepiphyseal dysplasia involving trabecular parts of long bones and the spine. The clinical syndrome was originally described independently by Drs. Luis Morquio and James Brailsford in 1929 [2]. The clinical, biochemical and molecular genetic features are summarized in Table 1.

## 2. Dysostosis Multiplex in MBD

The typical skeletal features of MBD include kyphoscoliosis, pigeon chest (pectus carinatum), a short neck, a large-appearing head with midface hypoplasia and mandibular protrusion, large-appearing joints (elbows, wrists, knees, ankles), coxa and genua valga, and flat feet. Joint laxity and tracheal stenosis are additional findings. Radiological findings include platyspondyly and vertebral beaking, odontoid hypoplasia, spinal canal narrowing, hip dysplasia, dysplasia of the carpal and tarsal bones, as well as shortening and epi-and metaphyseal dysplasia of long bones (e.g., shortening of the ulna and sloping of the distal ends of the radius and ulna) [3].

Short stature with a disproportionally short trunk is a constant feature. Growth deficiency develops progressively with increasing age. Whereas younger children’s height is still within the normal range, body heights in adults are significantly below the third percentile [3]. According to our recently performed online survey among patients with MBD, the self-reported mean weights and heights of the majority of participants over 19 years of age were far below the 15th percentile. Yet, compared to the average heights of patients with Morquio A disease, the MBD participants were considerably taller [4].

The structural and degenerative changes of bone, cartilage and connective tissue have numerous consequences on skeletal functionality and quality of life. Among the participants in our online patient survey, pain in joints and limbs, frequently associated with physical activity, along with mobility issues and limitations in self-care activities, were the most frequently reported challenges [4]. The majority of respondents were using walking aids by adolescence. Moreover, an average of three orthopaedic surgeries per person was reported, with hip and knee replacement most frequently performed in the second decade of life.

## 3. Other Organ and System Involvements

MBD patients also self-reported sleep disturbances, eye and dental problems, and heart and pulmonary disorders [4]. Corneal clouding and cardiac valve pathology are reported in 39% of 51 published cases [3]. Notably, corneal clouding was observed mainly in patients who had mutations in the catalytic domain essential for keratan sulfate substrate processing, such as W273L and Y333C [3]. Hepato-splenomegaly is a rare occurrence, described in a fraction of published cases [3]. Abnormal results of pulmonary function tests were reported by 40% of respondents to our online patient survey, with assessments done at a mean age of 20.4 years [4]. Another study applying a battery of pulmonary function tests in 4 individuals with MBD and 18 individuals with Morquio A disease concluded that patients with Morquio syndrome have small but normal functioning lungs, while restriction is posed by the chest’s structural abnormalities [5]. Tracheal obstruction with growth imbalances, short neck and adeno-tonsillar hypertrophy also contribute to airway-related problems [6]. Auditory alterations are common in mucopolysaccharidoses [7], though there were no reports in MBD specifically.

Neuronopathic manifestations including dystonia, dysarthria, dysphagia, ataxia, cognitive delay and epilepsy have been described in addition to skeletal features in some but not all patients with MBD. In order to distinguish both subtypes of MBD, patients with a combination of Morquio-like dysostosis and neuronopathic features should be classified as having *MBD plus*, while patients with isolated occurrences of Morquio-like dysostosis are classified as having *pure MBD* [3].

Spinal cord compression as a result of spinal canal stenosis is also common in adults. A recent review of 14 MBD cases reported spinal cord compression as a secondary neurologic manifestation due to spinal vertebral dysostosis in six patients with *MBD plus* and one patient with *pure MBD* [8]. Bladder incontinence due to spinal cord compression was observed in one individual [8].

## 4. Overlaps of MBD with Morquio A and GM1 Gangliosidosis

Systemic skeletal dysostosis is a common clinical presentation in both MBD and Morquio A disease. Despite these similarities, the degree of dysostosis is milder in MBD compared to Morquio A disease. Only mild Morquio A variants are clinically indistinguishable from MBD [8,9,10]. Despite intriguing clinical similarities, these two conditions actually arise from mutations in different genes. Morquio A disease is caused by alterations in the *GALNS* gene, resulting in deficient activity of the enzyme N-acetylgalactosamine-6-sulfatase (*GALNS*) and the subsequent accumulation of keratan sulfate and chondroitin-6-sulfate in lysosomes.

On the contrary, both Morquio B and GM1 gangliosidosis arise from mutations in the *GLB1* gene. Depending on the location of the mutations in the *GLB1* gene and on their combination in compound heterozygous individuals, the molecular pathophysiology of the resulting β-galactosidase protein can produce a spectrum of phenotypic presentations, ranging from primarily neurologic manifestations in GM1-gangliosidosis, to primarily skeletal involvement in MBD. The clinical heterogeneity of *GLB1*-related phenotypes may pose difficulties for disease nomenclature, thus obscuring research results, especially in cases of MBD. For example, in existing research studies, MBD cases are typically combined either with other mucopolysaccharidoses, such as Morquio A disease, due to their similarities in clinical presentation, or MBD and GM1 gangliosidosis, especially the late onset (adult) type, which are grouped together due to their sharing of similar neuronopathic elements.

## 5. *GLB1* Variants Associated with MBD

**Figure 1:**
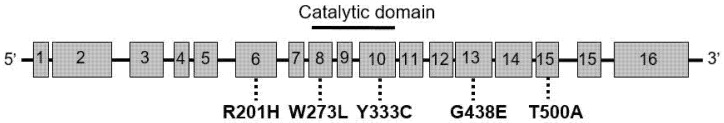
Structure of human *GLB1* gene and Morquio B (MBD)-related mutations.

The *GLB1* gene contains 16 exons spanning more than 60 kb (Figure 1). The longest transcript variant (NM_000404.2) is a 2.5 kb mRNA giving rise to a 70 kDa precursor protein which is processed within the lysosomes into the 64-kD mature β-galactosidase enzyme protein [11,12]. *GLB1* alternatively gives rise to a 2.0 kb mRNA transcript, formed by splicing out exons 3, 4 and 68, which encodes the elastin binding protein, a keyrecycling chaperone in the tropoelastin assembly process for elastogenesis in the extracellular matrix [13].

Mutations associated with type 1/infantile onset GM1-gangliosidosis, for the most part, are located in the core protein region, causing β-galactosidase instability, whereas mutations associated with milder phenotypes, such as types 2 and 3 GM1-gangliosidosis, tend to be on the protein surface [12]. W273L and T500A are the most frequently observed *GLB1* variants in patients with a Morquio-like dysostosis multiplex [3,8].

W273L is consistently associated with MBD-related skeletal dysostosis without neurological involvement (or *pure MBD*) [3], and could also serve as a predictor of the Morquio B phenotype [14]. W273L occurs in a highly conserved region of the *GLB1* gene where the amino acid residue Trp-273 resides at the entrance of the ligand-binding pocket, which acts as a holder of substrates for catalytic reactions. W273L affects the degradation of keratan sulfate more severely than the turnover of GM1-ganglioside, explaining the predominance of skeletal manifestations [12,15].

T500A is the second most prevalent allele that has been observed in compound heterozygosity in patients presenting with Morquio-like dysostosis multiplex with or without neuronopathic manifestations [3,8]. T500A is a missense mutation causing the premature degradation of β-galactosidase. While T500A has not yet been reported in homozygosity, evidence shows that compound heterozygosity is associated with abnormal keratan sulfate excretion, explaining its association with Morquio-like dysostosis multiplex.

An individual case homozygous for R210H was reported as *pure MBD*, however this association remains to be confirmed in more cases [16].

Homozygous G438E and Y333C were associated with *MBD plus* in single cases [3]. G438E causes an abnormal complex formation alone or when coupled with keratan sulfate binding [17] with a relatively high residual activity (6.1%) [18]. The results of enzyme activity assays using different natural substrates suggest that Y333C, similar to W273L, affects the active site of β-galactosidase rather than enzyme stability [19]. Y333H is not invariably associated with MBD, as homozygous cases have been described with type 2 (juvenile) GM1-gangliosidosis lacking the specific features of Morquio syndrome [18].

## 6. Biomarkers

Whereas in GM1 gangliosidosis the main accumulating substrates (GM1 and GA1 gangliosides) affect the central nervous system, in MBD, there is a preponderance of the accumulation of keratan sulfate in bones and cartilage. It is challenging to decipher whether, in humans, the abnormal chondrogenesis occurs in utero or after birth in MBD. A recent study of a CRISPR-Cas-edited murine Morquio B model harboring W273L failed to show an observable skeletal MBD phenotype in one-year-old mice, possibly due to the absence of keratan sulfate accumulation [20].

A correlation between residual β-galactosidase activities and MBD phenotypes has not been established [3]. This is most likely due to the use of synthetic substrates (e.g., 4-MU-β-galactoside) that only allow a rough discrimination between zero residual activities (e.g., infantile GM1-gangliosidosis) and activities up to 2–10% (e.g., late onset GM1-gangliosidosis and MBD) [18]. To precisely determine the biochemical characteristics of β-galactosidase mutants, measurements using natural substrates are needed. However, such measurements are laborious and have rarely been performed [21,22].

Likewise, there is little evidence showing a correlation between genotype and chemical biomarkers. Keratan sulfate is the main storage product in MBD, yet the traditional analytical methods of past publications have resulted in keratan sulfate either not being determined, or information about it being mostly restricted to its presence or absence. Quantitative measurements of keratan sulfate using LC-MS/MS-based technologies have only recently become available [23].

## 7. Cytokines

Emerging but limited evidence suggests cytokines may play a role in the pathophysiology of inflammation and pain in patients with MBD. A study of the in vitro MPS cells and animal models suggested that intracellular glycosaminoglycan storage can potentially trigger and maintain the inflammatory response via apoptosis of connective tissue cells, cartilage destruction, subsequent elevation of pro-inflammatory cytokines (predominantly TNF-alpha, IL-1β, and inflammatory proteases), or the activation of the TLR4 pathway leading to the release of TNF-alpha and IL-1β [24,25]. A recent study on pro-inflammatory cytokines in MPSI VA, MPS II and MBD found that, in comparison to normal controls, the levels of IL-6 and TNF-α were significantly elevated in untreated MBD patients (levels were similar to ERT-treated MPS IVA, but lower than untreated MPS IVA patients). Notably, this study focused on MPS IVA (*n* = 34) with only five MBD participants, allowing for limited analysis and conclusions [26].

## 8. Treatment

Currently for MBD, supportive and symptomatic therapy is the only currently available treatment option, with orthopedic surgeries being the mainstay of therapies [4].

In recent decades, several approaches have been explored to find effective causal therapies for lysosomal storage diseases, including enzyme replacement therapy, substrate reduction therapy, hematopoietic stem cell transplantation therapy, pharmacological chaperone therapy and gene therapy. In the case of *GLB1*-related conditions, gene therapy is becoming available for type 1 and type 2 GM1-gangliosidosis, and is currently in phase 1–2 of clinical trial [27]. Enzyme replacement therapy and substrate reduction therapy with miglustat were also explored, particularly for brain pathologies of GM1-gangliosidosis in animal models [28] and in clinical case studies [29]. There still remains controversy as to whether these approaches are effective therapeutic interventions for the bone disease in *GLB1*-related MBD. Compared to other approaches, pharmacological chaperone therapy with small molecules shows advantages in terms of oral administration and broad tissue distribution, and potential compounds were developed for the treatment of GM1-gangliosidosis and MBD [30]. In addition, the synergetic effect of the pharmacological chaperone with other therapeutic approaches has emerged as a new option for future therapy [31].

## 9. Research

Knowledge of the natural history and genotype–phenotype correlation of *GLB1*-related conditions associated with the various types of dysostosis multiplex are essential to establishing appropriate nomenclature for *GLB1*-related dysostoses, and to inform the design and choice of outcomes in future clinical trials. For this purpose, our team has developed a patient-reported registry focusing on MBD, as well as a multi-center database for the longitudinal clinical monitoring of patients with *GLB1*-related dysostosis, and the biobanking of biological samples is currently being implemented by the team at the University of British Columbia https://clinicaltrials.gov/ct2/show/NCT04320329.

## Figures and Tables

**Table 1:** Summary of the clinical, biochemical, and genetic features of Morquio B disease.

System/Feature	Symptoms/Signs
Skeletal	Kyphoscoliosis, short trunk, pectus carinatum, short neck, coxa and genua valga, flat feet, joint laxity, and progressive growth impairment.
Radiological	Platyspondyly, odontoid hypoplasia, spinal canal narrowing, hip dysplasia, dysplasia of the carpal and tarsal bones, shortening and epi- and metaphyseal dysplasia of long bones.
Neurological (MBD plus)	Dystonia, dysarthria, dysphagia, ataxia, cognitive delay and epilepsy, spinal cord compression due to spinal canal narrowing.
Other organs	Corneal clouding, cardiac valve pathology, tracheal stenosis, adeno-tonsillar hypertrophy, dental problems. Hepatosplenomegaly is rare.
Functional outcomes	Pain in joints and limbs, limited mobility, limitation in self-care activities, abnormal pulmonary function (restrictive/obstructive lung disease, mainly due to chest structural abnormalities), sleep disturbances.
Reported surgeries	On average 3 orthopedic surgeries reported by the second decade of life, most frequently hip and knee replacement.
Biochemical	Accumulation of keratan sulfate in bone and cartilage, elevation of TNF-alpha, IL-1β, IL-6 and inflammatory proteases.
β-galactosidase activity	From 2 to 11.5% (pure skeletal presentation), and from 4.6 to 14.1% (MBD with neuronopathic presentation)
Reported *GLB1* mutations	W273L, T500A, R210H, G438E, Y333C, G438E, T384S, Y333H (not invariably associated with MBD).
